# Minitraps: A simple, compact, low-cost, and reusable method for collecting soil nematodes.

**DOI:** 10.17912/micropub.biology.000802

**Published:** 2023-04-17

**Authors:** Rebekah J White, Lara Oliveira Clemente

**Affiliations:** 1 Department of Biosciences, University of Exeter, Exeter, England, United Kingdom; 2 PPG Ecologia, Universidad Federal de Viçosa, Viçosa, MG, Brazil; 3 Iracambi Research Center, Muriaé, MG, Brazil

## Abstract

Soil organisms are a crucial part of the terrestrial biosphere and are essential for ecosystem functioning. A major part of soil and sediment ecosystems are nematodes worms, which can be used as a bioindicator of soil status. These worms represent one of the most numerous animal phyla on earth, filling all trophic levels in the soil food web. Overall nematode abundance is related to net ecosystem productivity, and regional variations in abundance provides insight into local patterns of soil fertility and functioning.

Methods for extracting nematodes from soils have been established, but these can be cumbersome, or require specialist equipment or consumables, meaning they are not always suitable for the field or remote areas. We have built on previous methods to develop a simple, more compact, and zero-waste method of extracting nematodes, using basic equipment. We demonstrate this in a small collection of soils from deforested, native forest, and reforested sites. On a larger scale, this method can be used to reflect overall ecosystem function, indicating current soil status, and future success and proliferation of reforested sites.

**Figure 1.**
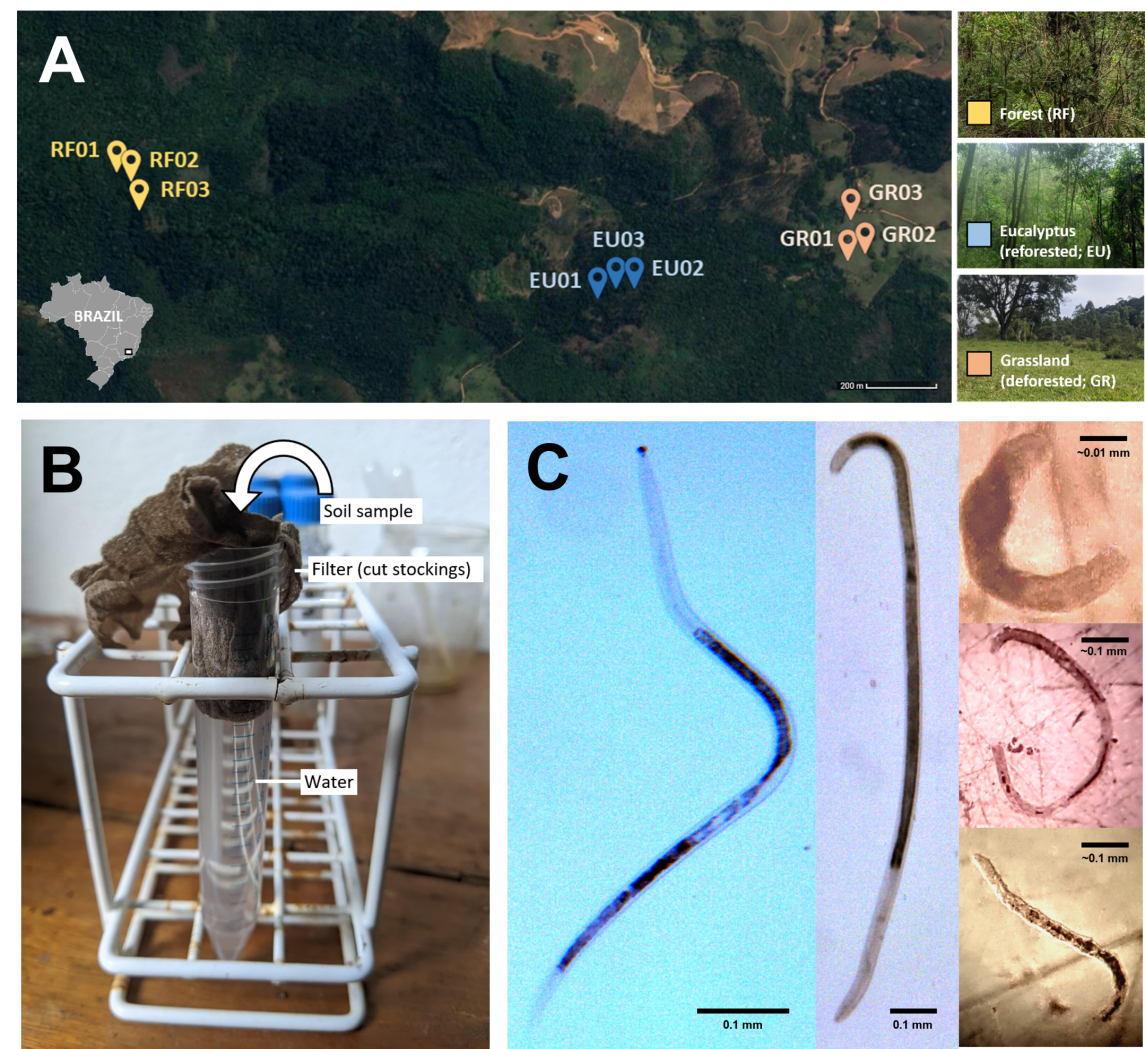
(a) Location of the study sites around Iracambi, Minas Gerais State, in the southeast of Brazil (modified from Google Earth). Right: photograph of characteristic vegetation of each study site (RF, EU and GR); (b) Microfauna minitrap setup using 15 ml Falcon tube and stockings; (c) Microfauna isolated from three subsystems. Left and middle: GR, image taken using Leica MC170 HD. Right: Images taken using Swift Instruments International Stereo M23 and smartphone. Right top: RF, likely plant parasitic *Meloidogyne* sp. third-stage juvenile. Right middle: RF, likely predatory *Tripyla* sp. Right bottom: GR, likely free-living *Alaimidae* family.

## Description

Soil organisms are a crucial part of the terrestrial biosphere and are essential for ecosystem functioning (Brussaard et al. 1997) . Despite this, there is often limited site-specific documentation of the impact of rewilding and reforestation programmes on biodiversity and ecosystem success in soil, such as in native woodland re-establishment (Burton et al. 2018) . Such studies can reflect overall forest ecosystem function, indicating future success and proliferation of recently reforested sites.

Nematodes can be used as a bioindicator of soil status, which has already been demonstrated in regions of Brazil (Caixeta et al. 2016, Cardoso et al. 2016) . These worms represent one of the most numerous animal phyla on earth, filling all trophic levels in the soil food web (Yeates 1987) . Overall nematode abundance is related to net ecosystem productivity, and regional variations in abundance provides insight into local patterns of soil fertility and functioning (van den Hoogen et al. 2019) .

Adapting from previous methods (van Bezooijen 2006a, Caixeta et al. 2016, Tintori et al. 2022) we present a simple, fast, and cheap method of extracting nematodes: the microfauna minitrap. It is simple to assemble, and the only specialist equipment required is basic and all reusable in this context: Pasteur pipettes, microscope, petri dishes, and 15 ml Falcon tubes. Additionally, analysing multiple small samples instead of fewer large samples has statistical advantages and provides more information about location or distribution of nematodes in a select subsystem (van Bezooijen 2006b). Indeed, previous studies have indicated that nematode variance between samples is “always greater than within a single sample” — so to avoid subsample bias, it is beneficial to take many samples / replicates rather than fewer large samples (van Bezooijen 2006b). Use of minitraps mitigates this issue by processing multiple smaller soil collections. Here, to demonstrate, we apply the microfauna minitrap method to extract soil nematodes from a small sample of native rainforest (RF), eucalyptus-reforested (EU), and grass (GR) soils.

**Methods**

Table 1. Equipment list for soil collection and microfauna minitraps

**Table:** 

**Purpose**	**Equipment**	**Source for this study**
Collection of soil samples	Metal spatula	Any
	Ziplock bags	Any – can be handwashed and reused
	GPS recorder (or smartphone with satellite signal capabilities)	Smartphone with Google Maps
Microfauna minitrap	15 ml Falcon tubes	Grenier Bio-One cat. no. 188271 – can be gently washed and reused (avoid scratching)
	Mesh material for filtering	Stockings cut to 10 x 10 cm – can be handwashed and reused with a pore size of ~20 µm
	Filtered water	On site
Collecting microfauna	Microscope x20 magnification	Leica MC170 HD or Swift Instruments International Stereo M23
	Pasteur pipettes	Grenier Bio-One cat. no. 612301
	Eyelash worm pick	(Hart 2006)
	Microscope camera or quality smartphone camera	Leica MC170 HD or smartphone (Google Pixel 3 G013A)
	Petri dish	Any – can be gently washed and reused (avoid scratching)

**Sampling sites**

For each subsystem (table 2), three sample sites were selected more than 1 metre away from an edge and at least 20 m away from each other ( figure 1a ). At each site, GPS location was recorded, and four soil subsamples were taken 1 metre north, east, south, and west. To collect soil, a metal spatula was used to dig ~1 inch below the surface, and a sample taken of 3 cm ^2^ . Ambient temperature and last rainfall were also recorded.

Table 2. Subsystems sampled in this study.

**Table:** 

**site**	**type**	**coordinates**	**vegetation**
EU01	Eucalyptus (reforested)	20°55'37.6"S 42°32'46.2"W	Mixed eucalyptus species planted >15 years ago.
EU02	Eucalyptus (reforested)	20°55'36.9"S 42°32'44.1"W	Mixed eucalyptus species planted >15 years ago.
EU03	Eucalyptus (reforested)	20°55'37.0"S 42°32'45.2"W	Mixed eucalyptus species planted >15 years ago.
GR01	Grassland (deforested)	20°55'35.4"S 42°32'25.3"W	*Brachiaria* grass field, very little other vegetation (< 5 trees / 100 m).
GR02	Grassland (deforested)	20°55'34.4"S 42°32'23.9"W	*Brachiaria* grass field, very little other vegetation (< 5 trees / 100 m).
GR03	Grassland (deforested)	20°55'32.1"S 42°32'25.3"W	*Brachiaria* grass field, very little other vegetation (< 5 trees / 100 m).
RF01	Rainforest	20°55'27.2"S 42°33'31.1"W	Native rainforest.
RF02	Rainforest	20°55'27.6"S 42°33'30.2"W	Native rainforest.
RF03	Rainforest	20°55'29.9"S 42°33'29.3"W	Native rainforest.

**Microfauna collection protocol**

1. Setting up microfauna minitrap

· Fill Falcon tube with 12 ml filtered or tap water.

· Add ~3 cm ^2^ damp soil sample to stocking filter.

· Place filled filter in Falcon tube and gently push down with spatula until submerged ( figure 1b ).

· Leave in a shaded, stationary area for 12-24 hours. Worms will move downward, through stockings, and into water.

2. Filtering soil

The sediment pellet will be at the bottom of the Falcon tube and may contain worms, so here we suggest carefully removing this first and examining under the microscope. One would expect a layer above the pellet to contain most/all the worms in the sample. We suggest the following workflow:

· Gently remove stocking filter.

· Slowly remove the clear supernatant to ~8 mL, and avoid disturbing pellet.

· Use the Pasteur pipette to remove sediment from the pellet and drop onto a petri dish. Observe under a microscope to check for worms. Denser sediment may require diluting with filtered water.

· When sediment pellet is gone, either gently centrifuge (if available) or manually check the tube under the microscope for further worms and use Pasteur pipette to move worms onto petri dish.

3. Collecting nematodes

· Label a new petri dish with sample site and add a drop of filtered water.

· Using a worm pick, move worms collected in step 2 into the water droplet.

· Photograph or image immediately, and take a video if possible (behaviour and motility may help with identification).

· Can be morphologically identified using Nemakey ( https://nematode.unl.edu/nemakey.htm ) and Mekete et al. 2012.

**Results**

All soil samples were taken in overcast conditions within 22-28°C ambient temperature, with heavy rainfall within the last 24 h. Using a small sample of three sites per subsystem, each run across three minitraps (resulting in 27 samples analysed), we collected 6 animals from RF soil, 6 from EU, and 7 from GR. While this method is therefore not suitable for quantitative studies which aim to collect every single nematode present, the worms isolated here were distributed across subsamples and can provide a rapid, low-cost qualitative result.

Nematodes collected were morphologically distinct ( figure 1c ), demonstrating the suitability of the method for isolating multiple species of different sizes. Morphology also suggests these nematodes belong to several trophic levels, including predatory, plant parasitic, and free-living soil individuals.

**Discussion**

Here we present a simple, low-budget, and reusable system for extracting nematodes from multiple trophic groups in soil. This method is particularly useful for remote areas, such as reforestation projects in Brazil’s forests. Additionally, this method is suitable for “on-the-go” extraction or for travelling, as most equipment required is all small in size. While this method does not combat the issue of quantitative accuracy – an issue that persists in currently used methods (Caixeta et al. 2016) – the use of several minitraps allows for higher number of replicates and less sensitivity to outliers and can provide more information about location or distribution of nematodes in a subsystem.

Full studies using this method should increase number of replicates (for a guideline, refer to Wiesel et al. 2015). To further this approach, extracted nematodes can be identified to the species level using DNA extraction molecular barcoding with SSU primers (Kenmotsu et al. 2021) . Trophic groups, such as from Yeates (1987), can also be assigned to each nematode family/genus identified. With these extensions, this method can be applied to determine to what extent eucalyptus and grassland environments are suitable for nematode populations compared to native rainforest sites, and therefore indicate soil status.

**Data Access Statement**

This study did not generate any new data.
